# Supporting Older Adults Using Long-Terms Services and Supports: Supporting Needs and Enhancing the Workforce

**DOI:** 10.1093/geroni/igaf122.1309

**Published:** 2025-12-31

**Authors:** Stephanie Giordano, Lindsay Dubois, Joe Caldwell

## Abstract

The demand for home- and community-based services (HCBS) continues to grow, due to population aging and increased desire to receive services in the community rather than in congregate settings. Despite this demand, there are still significant workforce shortages, contributing to unmet need and reliance on unpaid natural supports. The COVID-19 pandemic exacerbated and brought greater attention to the workforce shortages, while also requiring rapid innovation to the ways in which services were delivered. One important change to ensure older adults and people with disabilities were able to continue to get the services and supports they need was effectuated through Appendix K, allowing for family to be paid as caregivers. This symposium focuses on how service needs of older adults receiving long-term services have changed since COVID-19, the important role of family caregivers in support people to achieve good outcomes, and national benchmarks highlighting challenges direct service providers face in recruiting and retaining a stable workforce. These presentations not only provide a foundation for understanding the needs of older adults, from the perspective of people receiving services, but also highlight implications for ways to use data to accelerate policy and practice innovations to improve services and supports for older adults receiving long term services.

